# Current status and factors influencing kinesiophobia in patients with meniscus injury: a cross-sectional study

**DOI:** 10.1186/s13018-025-05498-5

**Published:** 2025-01-30

**Authors:** Faqiang Tang, Pan Xu, Cai Jiang, Xiaohua Ke, Dunbing Huang, Yaling Dai, Zhonghua Lin, Shizhong Wang

**Affiliations:** 1 The First Affiliated Hospital of Fujian Medical University, Fujian Provincial Hospital, Fuzhou, China; 2 School of Rehabilitation, Fujian University of Traditional Chinese Medicine, Fuzhou, China; 3 Shengli Clinical Medical College of Fujian Medical University, Fuzhou, China; 4 Rehabilitation Medicine Center, Fujian Provincial Hospital, Fuzhou, China; 5 Fuzhou University Affiliated Provincial Hospital, Fuzhou, China; 6 Fujian Provincial Center for Geriatrics, Fujian Provincial Hospital, Fuzhou, China; 7 Department of Complementary Medicine, University of Johannesburg, Johannesburg, South Africa; 8 Department of Rehabilitation Medicine, Shanghai Fourth People’s Hospital, School of Medicine, Tongji University, Shanghai, China; 9 The School of Health, Fujian Medical University, Fuzhou, China

**Keywords:** Meniscus injury, Knee pain, Kinesiophobia, Psychosocial factors

## Abstract

**Objectives:**

This study aimed to examine the relationships between kinesiophobia and injury severity, balance ability, knee pain intensity, self-efficacy, and functional status in patients with meniscus injuries and to identify key predictors of kinesiophobia.

**Design:**

A single-center, prospective cross-sectional study.

**Methods:**

A cross-sectional study involving 123 patients diagnosed with meniscus injuries at Fujian Provincial Hospital was conducted. The knee range of motion test was used to determine limitations in knee joint mobility, whereas magnetic resonance imaging (MRI) was used to assess the severity of meniscus damage. Several validated scales were administered: the Tampa Scale of Kinesiophobia (TSK-17) to measure kinesiophobia, the visual analog scale (VAS) to assess pain intensity, the general self-efficacy scale (GSES) to evaluate self-efficacy, and the Lysholm knee score (LKS) to assess knee functional status. Additionally, balance ability was assessed using the Huber 360 Neuromuscular Control Training and Assessment System (DJO, USA). Spearman’s correlation analysis was applied to explore factors associated with kinesiophobia, whereas simple linear regression analysis was used to identify its predictors.

**Results:**

Among the 123 participants included in the study, 60.16% were identified as experiencing kinesiophobia. Among these participants, 69.10% had grade III meniscus injuries, and 33.3% exhibited limited joint movement. The key clinical characteristics were as follows: the median VAS score was 4 (IQR 2–6), the GSES score was 22 (IQR 20–29), and the LKS score was 45 (IQR 38–55). Kinesiophobia was significantly correlated with injury severity, limited joint movement, pain intensity, self-efficacy, and other functional parameters (*P* < 0.05). However, no significant correlation was detected between kinesiophobia and limits of stability. Simple linear regression analysis (R²=0.917) revealed several significant predictors of kinesiophobia, including injury severity (β = 2.08), pain intensity (β = 0.882), Romberg quotient (RQ) (β = 3.239), and limited joint movement (β = 0.868). In contrast, self-efficacy (β =-0.455) was negatively associated with kinesiophobia. Furthermore, Grade III injuries and RQ were found to be associated with markedly higher levels of kinesiophobia.

**Conclusion:**

Kinesiophobia is strongly associated with knee injury severity, limited joint movement, RQ, pain intensity, and self-efficacy, which are key predictors. Clinical interventions should focus on these factors to enhance rehabilitation outcomes.

**Supplementary Information:**

The online version contains supplementary material available at 10.1186/s13018-025-05498-5.

## Introduction

Meniscus injury is a prevalent musculoskeletal condition that can lead to physical dysfunction across various age groups. With the widespread adoption of arthroscopy, approximately 4 million meniscus surgeries are performed globally each year, creating significant challenges for healthcare systems [1]. The annual incidence of meniscus injuries 70 per 100,000 individuals, with a significantly greater occurrence observed in individuals over the age of 40 [2]. Furthermore, the injury rate is even more pronounced in physically active populations, such as military personnel and athletes, particularly those involved in sports such as football, basketball, gymnastics, skiing, and wrestling [3, 4]. Meniscus injuries are typically caused by either acute trauma or chronic overuse [5], both of which can impair neuromuscular function. Local inflammation in damaged tissue increases the sensitivity of peripheral sensory neurons, resulting in repeated abnormal afferent signals to the central nervous system [6]. A previous study indicated that individuals who have undergone meniscectomy and returned to sports experience elevated levels of kinesiophobia [7]. This fear of movement is also commonly observed in patients with other knee conditions, including anterior cruciate ligament reconstruction, patellofemoral pain, and knee osteoarthritis [8, 9].

Kinesiophobia is a condition characterized by an irrational fear of excessive body movement or activity due to concerns about pain, injury, or reinjury. This fear often leads individuals to avoid the training and exercise essential for the recovery of knee joint function, ultimately hindering their rehabilitation [10, 11]. Following a knee injury, kinesiophobia typically worsens and is closely associated with a reduced quality of life [12]. Additionally, increased levels of kinesiophobia can delay recovery, impede the return to sports, and negatively affect muscle activity and motor strategies [13–15]. Research has indicated that in women with patellofemoral pain, kinesiophobia contributes to abnormal knee joint movement patterns [13]. A recent study suggested that kinesiophobia could influence neuromotor processes and cortical motor pain responses, thereby perpetuating pain and obstructing recovery [16]. In patients with meniscus injuries, the fear of movement is often driven primarily by an irrational psychological fear of overexertion or physical activity. This psychological fear is exacerbated by symptoms such as pain, limited joint movement, and muscle weakness, which further contributes to the development of kinesiophobia. Both psychological and physiological factors interact to form a complex barrier to physical activity in this patient population. Therefore, examining the factors associated with kinesiophobia in individuals with meniscus injuries provides a deeper understanding of their condition, which could lead to the development of personalized rehabilitation programs aimed at optimizing clinical outcomes for this group [17]. This study investigated the relationships between kinesiophobia induced by meniscus injury and injury severity, balance ability, knee pain, self-efficacy, and functional status. We hypothesized that kinesiophobia would be significantly correlated with injury severity, balance ability, knee pain, self-efficacy, and functional status. Additionally, we explored whether these factors could serve as predictors of kinesiophobia.

## Methods

### Study design

This was a single-center, prospective cross-sectional study conducted at Fujian Provincial Hospital from April 2023 to December 2023. This study was conducted in accordance with the Declaration of Helsinki and was approved by the Medical Ethics Committee of Fujian Provincial Hospital (No. K2023-03-041, Date of approval: March 28, 2023). This study was registered at the Chinese Clinical Trial Registry (No. ChiCTR2300073365). All patients provided written informed consent.

### Sample size calculation

Sample size calculations were conducted using G*Power 3.1 software (Düsseldorf, Germany) [18]. A minimum of 90 participants were required for multiple linear regression analysis with 17 potential predictors, a significance level of 0.05, a 95% confidence interval, and a medium effect size (F² = 0.15). To account for potential dropouts and ensure adequate statistical power, an additional 33 participants were recruited, resulting in a final sample size of 123 participants.

### Participants

The inclusion criteria were as follows: participants had a clinically confirmed diagnosis of unilateral meniscus injury, demonstrated clear consciousness, and possessed adequate cognitive and communication abilities to comply with the study procedures.

Exclusion criteria: pregnancy; concomitant anterior cruciate ligament injury or other ligament injuries; significant flexion contracture deformity in the injured anterior knee joint; additional musculoskeletal conditions, such as ankle sprains, hip impingement syndrome, or trunk pain (e.g., nonspecific low back pain); limb instability caused by nontraumatic factors; neurological or vestibular disorders, including stroke, Parkinson’s disease, vestibular neuritis, or other conditions affecting balance; inability to stand on one leg; previous treatment for meniscus injury; or withdrawal from the study prior to completion.

### Measurements

#### Data collection

Trained researchers communicated with eligible patients prior to the study and completed a general information questionnaire after admission. The questionnaire collected data on age, body mass index (BMI), sex, education level, and duration from injury to assessment. Subsequently, evaluations were conducted, including range of motion (ROM) testing of the affected knee joint and assessments using the Tampa Scale of Kinesiophobia (TSK-17), magnetic resonance imaging (MRI), balance ability, the visual analog scale (VAS), the general self-efficacy scale (GSES), and the Lysholm knee score (LKS). The questionnaires were collected and meticulously reviewed onsite. Patient data were gathered and organized by two researchers on the basis of electronic medical records. All the investigators held at least a bachelor’s degree in medicine and received training before participating in the assessment.

#### Limited joint movement

Knee ROM was measured following American Academy of Orthopedic Surgeons (AAOS) standards [19]. In the lateral decubitus position, the patient flexed the affected knee to the maximum range, and the goniometer was positioned with the axis at the lateral femoral epicondyle, the stationary arm aligned with the femur, and the moving arm aligned with the fibula. The flexion ROM was recorded after gentle pressure was applied at the end range, with angles outside 120–150° classified as restricted flexion. For extension, the patient fully extended the knee or as far as possible, with ROM measured similarly. Patients unable to reach 0° were classified as having restricted extension (-10°–0° considered normal). If either flexion or extension fell outside the normal range, the joint was classified as having restricted ROM.

#### Fear of movement

The TSK-17 was used to assess patients’ fear of movement. The scale consists of 17 items, each rated on a 4-point Likert scale ranging from 1 to 4. The total score ranges from 17 to 68, with a score > 37 indicating the presence of kinesiophobia in the patient [20].

#### Meniscus damage

MRI was performed using a Siemens 3.0 T scanner with T1- and T2-weighted sequences in the axial, coronal, and sagittal planes. An experienced radiologist evaluated the meniscus condition on the basis of standardized grading criteria: (1) Degree of damage: Grade I, punctate or small patchy high-signal intensity within the meniscus; Grade II, linear high-signal intensity not extending to the meniscal surface; Grade III, linear high-signal intensity reaching the superior or inferior surface [21].

#### Balance ability

The Huber 360 neuromuscular control training and evaluation system (DJO, USA), which was used to assess balance ability in participants, has been validated in previous studies [22, 23]. A fixed frame was installed on the platform, and the participants stood barefoot with the inner edges of their feet against the sides of the frame, with their arms hanging naturally at their sides. They were instructed to maintain an upright posture, with their head and chest raised and eyes facing forward, following system prompts to complete the test. Prior to the assessment, a single familiarization session was conducted to ensure that the participants understood how to use the equipment and perform the test correctly, minimizing performance variability due to unfamiliarity with the procedure and enhancing the reliability of the assessment.

The testing protocol involved several tasks performed sequentially: standing with one’s eyes open for 50 s, standing with one’s eyes closed for 50 s, standing on one’s foot with one’s eyes open for 30 s, and shifting the center of gravity (COG) in response to directional arrows displayed on a screen. Movements were made in eight randomized directions (forward, backward, left, right, and diagonal), with participants returning to the center between directions. The tests were conducted by experienced therapists, and the following key parameters were measured to evaluate balance control: (1) balance ability: track length (LNG), which represents the total COG sway path length, where longer paths indicate poorer COG control; the Statokinesigram area (SSKG), which reflects the range of COG sway, with larger areas suggesting more severe balance impairments; sway velocity (speed), where higher velocities indicate faster, less controlled COG movements; the Romberg quotient (RQ), which is the ratio of SSKG under eyes-closed to eyes-open conditions, indicating the compensatory role of vision in balance; and limits of stability (LOS), which reflects the ability to shift the COG while maintaining postural stability. These metrics provide a comprehensive assessment of balance control and postural stability.

#### Pain intensity

Pain intensity in patients with meniscus injury was assessed using the VAS. The scale ranges from 0 to 10, where 0 indicates no pain, 1–3 represents mild pain, 4–6 indicates moderate pain, 7–9 corresponds to severe pain, and 10 signifies intense pain. A higher score reflects greater pain intensity in the patient [24].

#### Self-efficacy

The GSES was used to assess patients’ self-efficacy. It consists of 10 items, each rated on a 4-point scale ranging from 1 to 4. The total score ranges from 1040, with higher scores indicating greater self-efficacy in the patient [25].

#### Knee functional status

Knee function status was assessed using the LKS, which includes 8 items: limp, support, locking, pain, instability, swelling, stair climbing, and squatting. The total score ranges from 0 to 100, with higher scores indicating better knee function [26].

### Statistical analysis

Statistical analyses were conducted using IBM SPSS Statistics version 26.0. The normality of the variables was assessed with the Shapiro‒Wilk test. Continuous variables that followed a normal distribution are presented as the means ± standard deviations (SDs), whereas nonnormally distributed variables are expressed as the medians with interquartile ranges (IQRs). Categorical variables are reported as the frequencies (*n*) and proportions (%). Spearman’s correlation analysis was performed to examine the relationships between kinesiophobia and its potential influencing factors. The correlation coefficients were interpreted as follows: coefficients of 0.2 or less indicated weak correlations, coefficients between 0.3 and 0.5 indicated moderate correlations, coefficients between 0.6 and 0.7 represented moderate to strong correlations, and coefficients of 0.8 or higher signified strong correlations [27]. Furthermore, multivariable linear regression analysis was used to derive unstandardized regression coefficients (β). The predictive power of each final model was assessed by calculating the R-squared (R²) value, which indicates the percentage of variance explained. This analysis aimed to determine whether injury severity, limited joint movement, RQ, pain intensity and self-efficacy were significant predictors of kinesiophobia.

## Results

### Participant characteristics

**Fig. 1:**
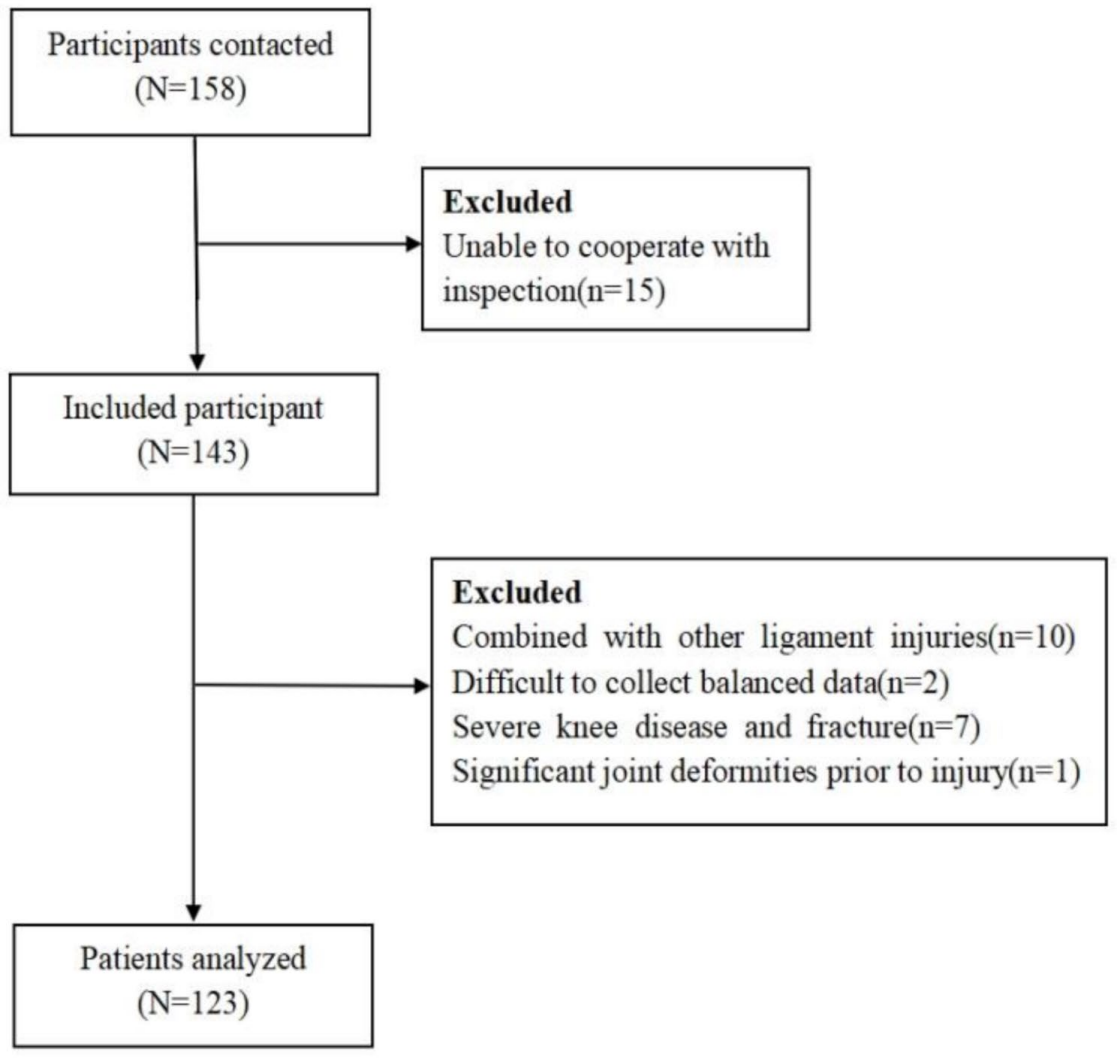
Recruitment flow chart

Intotal, 158 potential patients were initially contacted; however, 15 patients were unable to visit the hospital for the examination due to business commitments. Additionally, 20 patients did not meet the inclusion criteria and were therefore excluded from the study (Fig. 1).

Table 1 presents the baseline characteristics of the study participants. The median age was 53 years (IQR: 44–61), and the mean BMI was 24.84 ± 3.09 kg/m², with a range from 18.37 to 31.25 kg/m². Among the participants, 74 (60.2%) were female, and 30 (24.39%) had attained a college degree or higher. The median duration between injury and assessment was 9 weeks (IQR: 2–16). Additionally, 85 (69.10%) patients had Grade III injuries, and 41 (33.3%) had restricted joint mobility.

**Table 1:** Baseline characteristics of the study participants (*n* = 123)

Variables	Value
Age(years), median (IQR)	53(44–61)
BMI(kg/m^2^),mean (SD)	24.84 ± 3.09
Sex	
Male, (*n*/%)	49(39.80)
Female, (*n*/%)	74(60.20)
Highest level of education	
Primary, (*n*/%)	36(29.27)
Secondary, (*n*/%)	28(22.76)
Senior, (*n*/%)	29(23.58)
Junior college, (*n*/%)	19(15.45)
Bachelor’s degree or above, (*n*/%)	11(8.94)
The duration from injury to assessment(month)median (IQR)	9(2–16)
Damage degree	
< III degree, (*n*/%)	38(30.90)
III degree, (*n*/%)	85(69.10)
Limited joint movement	
unrestricted, (*n*/%)	82(66.7)
limitation, (*n*/%)	41(33.3)

BMI, body mass index

Over 60% of the participants (60.16%) were found to experience kinesiophobia. The clinical characteristics of the subjects are presented in Table 2, which includes the TSK-17 scores, VAS scores, GESE scores, and Lysholm knee scores. The median TSK-17 score was 41 (range: 32–46), indicating a moderate level of kinesiophobia. For pain assessment, the median VAS score was 4 (IQR: 2–6). The GESE score, which reflects the general evaluation of knee function, had a median value of 22 (IQR: 20–29). Finally, the Lysholm knee score, which reflects knee-specific function and symptoms, had a median of 45 (IQR: 38–55).

**Table 2:** Clinical characteristics of the participants

Variables	Value
TSK-17(17–68),median (IQR)	41(32–46)
VAS(0–10),median (IQR)	4(2–6)
GSES(10–40),median (IQR)	22(20–29)
LKS(0-100),median (IQR)	45(38–55)

TSK-17, Tampa Scale for Kinesiophobia-17; VAS, visual analog scale; GES, general self-efficacy scale; LKS, Lysholm knee score

Table 3 presents the balance ability of the participants under various conditions. When standing on the platform with both feet and eyes open, the mean LNG was 690.72 ± 143.97 mm, with a range of 408.01–1120.33 mm. The median SSKG value (IQR) was 316.29 (212.85–430.74) mm², and the mean speed was 13.82 ± 2.88 mm/s, with a range of 8.16–22.41 mm/s. In contrast, when standing with both feet and eyes closed, the median LNG value (IQR) was 987.89 (837.10-1142.83) mm, whereas the median SSKG value (IQR) was 532.63 (395.11-634.32) mm². The median speed (IQR) was 19.76 (16.74–22.86) mm/s. For the affected leg with eyes open, the median LNG value (IQR) was 1533.58 (1265.21–1869.26) mm, and the median SSKG value (IQR) was 788.94 (537.08–1048.52) mm². The average LOS was 56697.00 ± 24536.13, with a range of 12966.22-114683.20. Additionally, the median RQ value (IQR) was 1.53 (1.17–2.40).

**Table 3:** Balance ability of participants

Variables	Value
Both side leg with eyes opening	
LNG(mm), mean (SD)	690.72 ± 143.97
SSKG(mm²), median (IQR)	316.29(212.85-430.74)
Speed(mms), mean (SD)	13.82 ± 2.88
Both side leg with eyes closed	
LNG(mm), median (IQR)	987.89(837.10-1142.83)
SSKG(mm²), median (IQR)	532.63(395.11-634.32)
Speed(mms), median (IQR)	19.76(16.74–22.86)
Affected side leg with eyes open	
LNG(mm), median (IQR)	1533.58(1265.21-1869.26)
SSKG(mm²), median (IQR)	788.94(537.08-1048.52)
LOS, mean (SD)	56697.00 ± 24536.13
RQ, median (IQR)	1.53(1.17–2.40)

LNG, tracklength; SSKG, statokinesigram area; LOS, Limits of Stability; RQ: Romberg Quotient

### Spearman’s correlation analysis of factors related to kinesiophobia

Spearman correlation analysis revealed several significant relationships, as summarized in Table 4. Kinesiophobia was significantly positively correlated with injury severity, limited joint movement, the RQ, the VAS score, the GSES score, track length, the statokinesigram area, speed with eyes closed, speed of the affected foot with eyes open, the Lysholm score, and the duration from injury to assessment. However, no significant correlation was detected between kinesiophobia and the length of hospital stay (LOS).

**Table 4:** Bivariate correlation analysis of factors related to kinesiophobia

Variables	*R*/Z	*P*
Damage degree	-7.515	< 0.001
Limited joint movement	-3.804	< 0.001
VAS	0.836	< 0.001
GSES	-0.855	< 0.001
LKS	-0.583	< 0.001
The duration from injury to assessment	-0.231	0.01
Both sides legs with eyes opening		
LNG	-0.419	< 0.001
SSKG	-0.471	< 0.001
Speed	-0.420	< 0.001
Both sides legs with eyes closed		
LNG	0.264	0.003
SSKG	0.529	< 0.001
Speed	0.271	0.002
Affected side leg with eyes open		
LNG	0.397	< 0.001
SSKG	0.614	< 0.001
LOS	-0.148	0.102
RQ	0.895	< 0.001

VAS, visual analog scale; GSES, general self-efficacy scale; SSKG, statokinesigram area; LKS, Lysholm knee score; LNG, tracklength; LOS, Limits of stability; RQ: Romberg Quotient

### Multifactor analysis of factors related to kinesiophobia among patients with meniscus injuries

The results of the simple linear regression analysis, presented in Table 5, yielded an R² value of 0.917. This finding indicated that the independent variables in the model (injury severity, limited joint movement, RQ, VAS score and GSES score) predicted 91.7% of the variance in kinesiophobia. The overall model fit was deemed satisfactory, as evidenced by an F value less than 0.05. Moreover, the variance inflation factor (VIF) was found to be less than 5, and the tolerance exceeded 0.20, confirming that there was no multicollinearity present in the model.

Specifically, the incidence of kinesiophobia in patients with Grade III injuries was approximately 2.080 units higher than that reported in patients with injuries below Grade III. Furthermore, for patients with meniscus injuries, those exhibiting restricted joint movement had a kinesiophobia score that was 0.868 units higher than that of patients with unrestricted joint movement. Additionally, each one-unit increase in the RQ and VAS scores was associated with increases in kinesiophobia of 3.239 and 0.882 units, respectively. In contrast, a one-unit increase in self-efficacy was linked to a decrease of 0.455 units in kinesiophobia.

**Table 5:** TSK-17 multiple linear regression (*n* = 123)

Model	Unstandardized Coefficients		Standardized Coefficients			95.0% Confidence Interval		Collinearity Statistics	
	B	Std. error	Beta	T	Sig.	Lower bound	Upper bound	Tolerance	Variance inflation factor
(Constant)	39.934	2.678		14.911	0.000	34.630	45.238		
Damage degree	2.080	0.609	0.130	3.415	0.001	0.874	3.287	0.471	2.123
Limited joint movement	0.868	0.432	0.055	2.011	0.047	0.013	1.724	0.900	1.111
RQ	3.239	0.403	0.361	8.038	0.000	2.441	4.037	0.337	2.969
VAS score	0.882	0.195	0.242	4.512	0.000	0.495	1.269	0.236	4.240
GSES score	-0.455	0.080	-0.308	-5.69	0.000	-0.614	-0.297	0.231	4.322

Dependent variable: TSK score. RQ, Romberg Quotient; VAS, Visual Analog Scale; GSES, General Self-Efficacy Scale

## Discussion

The results of this study indicate that kinesiophobia is positively correlated with injury severity, limited joint movement, RQ, and VAS scores, suggesting that as the degree of knee joint injury, joint movement limitations, RQ, and pain scores increase, the level of psychological fear also intensifies. Kinesiophobia is negatively correlated with self-efficacy, indicating that patients with lower self-efficacy experience higher levels of psychological fear. Moreover, injury severity, joint movement limitations, RQ, the VAS score, and the GSES score are significant predictors of kinesiophobia in patients.

In this study, individuals with meniscus injuries presented kinesiophobia, as measured by the TSK-17. A significant correlation was found between the degree of meniscus injury and the level of kinesiophobia. Additionally, the severity of meniscus injury was a positive predictor of kinesiophobia, indicating that more severe injuries were associated with higher levels of kinesiophobia. When the meniscus is damaged, its protective function is compromised, leading to reduced joint stability and an increased risk of secondary cartilage damage, which worsens the condition [28, 29]. A study reported that more severe meniscus injuries are linked to a greater likelihood of developing clinical symptoms [30]. For injuries below Grade III, the clinical symptoms are typically minimal and are often limited to localized edema or mild pain, which may not significantly impact participation in physical activities. However, when the injury reaches Grade III or higher, the tear becomes more severe, potentially affecting joint movement. In such cases, pain may occur during walking, or the injury may be accompanied by joint locking, knee snapping, or quadriceps atrophy, leading to restricted movement, abnormal gait, or even falls while walking [31]. Patients with severe meniscus injuries are more likely to develop a fear of movement to avoid pain or further injury. A similar study [32] also suggested that kinesiophobia is associated with knee joint pain, flexion, and overall function. Therefore, individuals with meniscus injuries should seek early diagnosis and treatment, gain a clear understanding of the extent of their injury, and follow medical advice to prevent further deterioration.

In this study, a significant correlation was found between RQ and kinesiophobia. Specifically, RQ was identified as a positive predictor of kinesiophobia in patients with meniscus injuries, meaning that higher RQ values were associated with higher levels of kinesiophobia. The RQ represents the ratio of static balance measured under eyes-closed conditions to that under eyes-open conditions [33]. A larger RQ value indicates a greater reliance on vision for maintaining static balance. When vision is controlled for and only proprioception and vestibular input remain, neuromuscular control becomes less effective in regulating static balance [34]. Balance is defined as the ability to maintain, achieve, or restore one’s position through postural adjustments [35], which depend on several factors, including biomechanical elements, spatial orientation, action strategies, dynamic control abilities, and sensory strategies [36–40]. Among these, sensory strategies, particularly those involving proprioceptive input, play crucial roles in postural control and cognitive processing [41, 42]. Proprioception, which refers to the detection of muscle contraction and joint position, provides essential feedback to the brain’s neurons for analyzing motor behavior. Consequently, proprioception is fundamental for maintaining body posture stability [43, 44]. Research has shown that mechanoreceptors in the anterior and posterior regions of the meniscus are involved in detecting proprioceptive signals, playing a critical role in the sensory feedback mechanism of the knee joint and its position regulation during movement [45]. Following a meniscus injury, impairments in proprioception reduce the ability to sense knee movement and select appropriate sensory inputs, weakening or even eliminating a patient’s perception of knee joint position, direction, and speed. This impairment contributes to the development of kinesiophobia. Similar studies have demonstrated a strong association between kinesiophobia and knee stability [46, 47]. Therefore, it is crucial for patients with meniscus injuries to gain an accurate understanding of their condition at an early stage, seek prompt medical evaluation, and follow medical recommendations to prevent the injury from worsening.

The findings of this study indicated that higher levels of pain were predictive of higher levels of kinesiophobia. Similar results have been reported in studies of other knee joint conditions [48, 49]. A patient’s fear of movement can be viewed as a coping strategy, potentially aimed at reducing knee joint pain. Pain is a subjective experience that involves both a physiological response and a complex perceptual process [50]. The perception and processing of pain involve multiple regions of the brain, including the prefrontal cortex, cingulate cortex, and amygdala, all of which contribute to both the sensory experience of pain and its emotional processing. As a result, external somatosensory inputs and internal motivational, affective, and cognitive processing may interact during the adaptive regulation of pain [51]. Previous studies have shown that pain intensity is correlated with pain catastrophizing, a phenomenon in which patients experience negative cognitive and emotional responses [52, 53]. This altered state of attention and expectation can intensify their emotional reactions to pain. Additionally, the tendency to catastrophize pain is linked to a higher incidence of chronic pain and muscle dysfunction [54]. According to the fear-avoidance model [55], catastrophic thinking following a pain episode leads to heightened kinesiophobia and increased sensitivity to pain. This creates a protective response in which patients adopt a negative coping strategy, often avoiding activities or movements that could trigger pain. Consequently, patients with higher pain levels may struggle to engage in regular rehabilitation exercises, resulting in increased kinesiophobia.

Some researchers have suggested that long-term fear of movement can lead to muscle fatigue and disuse atrophy, creating a vicious cycle that impairs functional performance [6, 16]. The results of this study revealed a moderate positive correlation between kinesiophobia and knee joint function scores. Patients with meniscus injuries may exhibit fear-avoidance behavior due to painful stimuli and reduced muscle strength, both of which can negatively impact lower limb functional performance. In a previous systematic review [56], Rethman proposed similar findings, stating that higher levels of kinesiophobia were associated with poorer functional performance. Furthermore, Smith reported that exercise therapy performed despite pain had certain benefits over exercises performed without pain [57]. This suggests that healthcare providers should promptly assess patients’ pain levels and implement appropriate interventions, particularly for those experiencing higher pain levels. By doing so, patients can gain a clearer understanding of the relationship between pain and exercise, alleviate their psychological distress, and engage in exercises within acceptable pain thresholds. Such an approach may help maximize the recovery of knee joint function.

A significant correlation between self-efficacy and kinesiophobia was found in this study, highlighting the considerable influence of self-confidence on the development of kinesiophobia. Self-efficacy was identified as a negative predictive factor for kinesiophobia in patients with meniscus injuries, suggesting that higher levels of self-efficacy are associated with lower levels of kinesiophobia [58]. Previous studies have also identified self-efficacy as one of the key psychosocial factors influencing meniscus injury rehabilitation [48, 59]. Self-efficacy refers to an individual’s confidence in their own abilities [60] and plays a crucial role in both the development of and response to kinesiophobia. Patients with low self-efficacy often fear that exercising will worsen pain or other physical symptoms, which can lead to feelings of helplessness and frustration. This, in turn, can intensify the avoidance of physical activity, resulting in poor adherence to early rehabilitation exercises and subsequently affecting treatment outcomes [61]. Ericsson reported that enhancing self-efficacy could help patients confront the stress associated with exercise-related fears in a positive and proactive manner [59]. Furthermore, increasing self-efficacy can aid patients in managing physical pain and emotional stress and facilitate the adoption of proactive coping strategies, such as participation in activities and functional exercises [62]. Similar findings were reported by Eliza in a study on musculoskeletal trauma [63]. Additionally, patients with high self-efficacy tend to demonstrate better compliance with treatment and achieve improved recovery outcomes. Therefore, healthcare professionals should implement interventions aimed at enhancing self-efficacy, such as educating patients on cognitive and behavioral pain management strategies through manuals and video tutorials and supporting them in mastering these techniques [64]. Assisting patients with low self-efficacy in building confidence to overcome their fear of exercise is essential, as this can encourage more active participation in pain management through exercise [65].

### Limitations

This study has several limitations that warrant attention. First, the cross-sectional design, while providing valuable insights into correlations, limited the ability to establish causal relationships. Longitudinal or experimental studies are needed to explore the directionality and causality of these associations. Second, the reliance on self-reported measures for pain intensity, self-efficacy, and kinesiophobia may have introduced biases due to variations in individual perceptions and emotional states. Third, the study did not account for other potentially significant clinical or biomechanical variables, such as preinjury activity levels, psychological conditions, or specific rehabilitation protocols. Additionally, the lack of detailed classification of meniscus damage (e.g., medial vs. lateral, anterior vs. posterior) may have masked important differences in how injury subtypes influence kinesiophobia. The focus on a single hospital also limits the generalizability of the findings, and a multicenter study with a more diverse sample is recommended. Moreover, the analysis of balance ability was limited, with insufficient exploration of specific deficits and their interaction with kinesiophobia. Socioeconomic and cultural factors, which significantly influence rehabilitation adherence and psychological responses, were also not considered. Finally, confounding variables, such as prior injuries or pharmacological interventions, were not controlled for, potentially impacting the observed relationships. Future research should adopt longitudinal designs; integrate objective measures; and consider additional clinical, demographic, and psychosocial variables to increase the robustness and applicability of the findings.

## Conclusion

This study demonstrated that kinesiophobia was significantly associated with knee injury severity, limited joint mobility, RQ, pain intensity, and self-efficacy, with these factors identified as key predictors. Patients with more severe injuries, greater joint mobility restrictions, and higher RQ and pain intensity exhibited higher levels of kinesiophobia, while higher self-efficacy was associated with reduced kinesiophobia. These findings underscore the multifaceted nature of kinesiophobia, driven by an interplay of physical and psychological factors, and highlight the importance of addressing these factors in clinical practice. The implications for clinical management are significant. Early identification and targeted interventions for kinesiophobia could mitigate its impact on rehabilitation outcomes. Clinicians should integrate strategies to enhance self-efficacy, address pain management proactively, and promote joint mobility and stability through tailored rehabilitation programs. Such approaches not only prevent the progression of kinesiophobia but also facilitate adherence to rehabilitation protocols, optimize recovery, and improve patients’ functional outcomes.

## Electronic supplementary material

Below is the link to the electronic supplementary material.

Supplementary Material 1

Supplementary Material 2

## Data Availability

No datasets were generated or analysed during the current study.

## References

[1] Hawker G, Guan J, Judge A, et al. Knee arthroscopy in England and Ontario: patterns of use, changes over time, and relationship to total knee replacement [J]. J Bone Joint Surg Am. 2008;90(11):2337–45. 10.2106/jbjs.G.01671 18978402 10.2106/JBJS.G.01671

[2] Fox AJ, Wanivenhaus F, Burge AJ, et al. The human meniscus: a review of anatomy, function, injury, and advances in treatment [J]. Clin Anat. 2015;28(2):269–87. 10.1002/ca.22456 25125315 10.1002/ca.22456

[3] Jones JC, Burks R, Owens BD, et al. Incidence and risk factors associated with meniscal injuries among active-duty US military service members [J]. J Athl Train. 2012;47(1):67–73. 10.4085/1062-6050-47.1.67 22488232 10.4085/1062-6050-47.1.67 PMC3418117

[4] Beals CT, Magnussen RA, Graham WC, et al. The prevalence of Meniscal Pathology in Asymptomatic athletes [J]. Sports Med. 2016;46(10):1517–24. 10.1007/s40279-016-0540-y 27075327 10.1007/s40279-016-0540-y

[5] Feehan J, Macfarlane C, Vaughan B. Conservative management of a traumatic meniscal injury utilising osteopathy and exercise rehabilitation: a case report [J]. Complement Ther Med. 2017;33:27–31. 10.1016/j.ctim.2017.05.007 28735822 10.1016/j.ctim.2017.05.007

[6] Yam MF, Loh YC, Tan CS, et al. General pathways of Pain Sensation and the major neurotransmitters involved in Pain regulation [J]. Int J Mol Sci. 2018;19(8). 10.3390/ijms19082164 10.3390/ijms19082164 PMC6121522 30042373

[7] Starbuck C, Walters V, Herrington L, et al. Knee offloading by patients during walking and running after meniscectomy [J]. Orthop J Sports Med. 2024;12(3):23259671231214766. 10.1177/23259671231214766 38524891 10.1177/23259671231214766 PMC10958822

[8] Tichonova A, RIMDEIKIENĖ I. The relationship between pain catastrophizing, kinesiophobia and subjective knee function during rehabilitation following anterior cruciate ligament reconstruction and meniscectomy: a pilot study [J]. Med (Kaunas). 2016;52(4):229–37. 10.1016/j.medici.2016.07.005 10.1016/j.medici.2016.07.005 27623044

[9] Bailey KM, Carleton RN, Vlaeyen JW, et al. Treatments addressing pain-related fear and anxiety in patients with chronic musculoskeletal pain: a preliminary review [J]. Cogn Behav Ther. 2010;39(1):46–63. 10.1080/16506070902980711 19697175 10.1080/16506070902980711

[10] Luque-Suarez A, Martinez-Calderon J, Falla D. Role of kinesiophobia on pain, disability and quality of life in people suffering from chronic musculoskeletal pain: a systematic review [J]. Br J Sports Med. 2019;53(9):554–9. 10.1136/bjsports-2017-098673 29666064 10.1136/bjsports-2017-098673

[11] De Vroey H, Claeys K, Shariatmadar K, et al. High levels of Kinesiophobia at Discharge from the Hospital May negatively affect the short-term functional outcome of patients who have undergone knee replacement surgery [J]. J Clin Med. 2020;9(3). 10.3390/jcm9030738 10.3390/jcm9030738 PMC7141217 32182895

[12] Goldberg P, Zeppier G, Bialosky J, et al. Kinesiophobia and its Association with Health-Related Quality of Life Across Injury locations [J]. Arch Phys Med Rehabil. 2018;99(1):43–8. 10.1016/j.apmr.2017.06.023 28760572 10.1016/j.apmr.2017.06.023

[13] De Oliveir Silva D, Barton CJ, Briani RV, et al. Kinesiophobia, but not strength is associated with altered movement in women with patellofemoral pain [J]. Gait Posture. 2019;68:1–5. 10.1016/j.gaitpost.2018.10.033 30408709 10.1016/j.gaitpost.2018.10.033

[14] Hart HF, Collins NJ, Ackland DC, et al. Is impaired knee confidence related to worse kinesiophobia, symptoms, and physical function in people with knee osteoarthritis after anterior cruciate ligament reconstruction? [J]. J Sci Med Sport. 2015;18(5):512–7. 10.1016/j.jsams.2014.09.011 25444950 10.1016/j.jsams.2014.09.011

[15] Leeuw M, Goossens ME, Linton SJ, et al. The fear-avoidance model of musculoskeletal pain: current state of scientific evidence [J]. J Behav Med. 2007;30(1):77–94. 10.1007/s10865-006-9085-0 17180640 10.1007/s10865-006-9085-0

[16] Duport A, Pelletier R, Martel M, et al. The influence of kinesiophobia and pain catastrophizing on pain-induced corticomotor modulation in healthy participants: a cross sectional study [J]. Neurophysiol Clin. 2022;52(5):375–83. 10.1016/j.neucli.2022.08.001 36220765 10.1016/j.neucli.2022.08.001

[17] Fuentes A, Hagemeister N, Ranger P, et al. Gait adaptation in chronic anterior cruciate ligament-deficient patients: pivot-shift avoidance gait [J]. Clin Biomech (Bristol Avon). 2011;26(2):181–7. 10.1016/j.clinbiomech.2010.09.016 20965627 10.1016/j.clinbiomech.2010.09.016

[18] Clayson PE, Carbine KA, Baldwin SA, et al. Methodological reporting behavior, sample sizes, and statistical power in studies of event-related potentials: barriers to reproducibility and replicability [J]. Psychophysiology. 2019;56(11):e13437. 10.1111/psyp.13437 31322285 10.1111/psyp.13437

[19] Russo RR, Burn MB, Ismaily SK, et al. Is digital photography an accurate and precise method for measuring range of motion of the hip and knee? [J]. J Exp Orthop. 2017;4(1):29. 10.1186/s40634-017-0103-7 28884315 10.1186/s40634-017-0103-7 PMC5589719

[20] Weermeijer JD, Meulders A, Clinimetrics. Tampa Scale for Kinesiophobia [J]. J Physiother. 2018;64(2):126. 10.1016/j.jphys.2018.01.001 29567379 10.1016/j.jphys.2018.01.001

[21] Stensby JD, Pringle LC, CRIM J. MRI of the Meniscus [J]. Clin Sports Med. 2021;40(4):641–55. 10.1016/j.csm.2021.05.004 34509203 10.1016/j.csm.2021.05.004

[22] He L, Chai SS, Chen YP. The Effect of Balance Training with Balance Assessment Training System on Balance after Stroke[J]. Chin J Rehabil Theory Pract. 2021;27(7):760–4.

[23] Markovic G, Sarabon N, Greblo Z, et al. Effects of feedback-based balance and core resistance training vs. pilates training on balance and muscle function in older women: a randomized-controlled trial[J]. Arch Gerontol Geriatr. 2015;61(2):117–23. 26036209 10.1016/j.archger.2015.05.009

[24] Sung YT, Wu JS. The visual analogue scale for rating, ranking and Paired-Comparison (VAS-RRP): a new technique for psychological measurement [J]. Behav Res Methods. 2018;50(4):1694–715. 10.3758/s13428-018-1041-8 29667082 10.3758/s13428-018-1041-8 PMC6096654

[25] Wilski M, Brola W, Koper M, et al. Relationship between physical activity and coping with stress in people with multiple sclerosis: a moderated mediation model with self-efficacy and disability level as variables [J]. Int J Clin Health Psychol. 2024;24(1):100415. 10.1016/j.ijchp.2023.100415 37840558 10.1016/j.ijchp.2023.100415 PMC10568286

[26] Qiao Y, Wu C, Wu X, et al. The value of minimal clinically important difference, substantial Clinical Benefit, and patient-acceptable symptomatic state for commonly used patient-reported outcomes in recurrent patellar instability patients after Medial Patellofemoral Ligament Reconstruction and Tibial Tubercle transfer [J]. Arthroscopy. 2024;40(1):115–23. 10.1016/j.arthro.2023.06.042 37419222 10.1016/j.arthro.2023.06.042

[27] Akoglu H. User’s guide to correlation coefficients [J]. Turk J Emerg Med. 2018;18(3):91–3. 10.1016/j.tjem.2018.08.001 30191186 10.1016/j.tjem.2018.08.001 PMC6107969

[28] Primeau CA, Birmingham TB, Leitch KM, et al. Degenerative meniscal tears and high tibial osteotomy: do current treatment algorithms need to be realigned? [J]. Clin Sports Med. 2019;38(3):471–82. 10.1016/j.csm.2019.02.010 31079775 10.1016/j.csm.2019.02.010

[29] Liu L, Xian Y, Wang W, et al. Meniscus-inspired self-lubricating and friction-responsive hydrogels for protecting articular cartilage and improving Exercise [J]. ACS Nano. 2023. 10.1021/acsnano.3c10139 37975685 10.1021/acsnano.3c10139

[30] Rohren EM, Kosarek FJ, HELMS C A. Discoid lateral meniscus and the frequency of meniscal tears [J]. Skeletal Radiol. 2001;30(6):316–20. 10.1007/s002560100351 11465771 10.1007/s002560100351

[31] Sullivan JK, Shrestha S, Collins JE, et al. Association between changes in muscle strength and pain in persons with meniscal tear and osteoarthritis [J]. Osteoarthr Cartil Open. 2020;2(3):100072. 10.1016/j.ocarto.2020.100072 36474676 10.1016/j.ocarto.2020.100072 PMC9718231

[32] Kocic M, Stankovic A, Lazovic M, et al. Influence of fear of movement on total knee arthroplasty outcome [J]. Ann Ital Chir. 2015;86(2):148–55. 25952608

[33] Van Parys JA, Njiokiktjien CJ. Romberg’s sign expressed in a quotient [J]. Agressologie, 1976, 17 specno: 95 – 9. 1008169

[34] Kalron A. The Romberg ratio in people with multiple sclerosis [J]. Gait Posture. 2017;54:209–13. 10.1016/j.gaitpost.2017.03.016 28346894 10.1016/j.gaitpost.2017.03.016

[35] Pollock AS, Durward BR, ROWE P J, et al. What is balance? [J]. Clin Rehabil. 2000;14(4):402–6. 10.1191/0269215500cr342oa 10945424 10.1191/0269215500cr342oa

[36] Liu Z, Wang Q, Sun W, et al. Balancing sensory inputs: somatosensory reweighting from proprioception to tactile sensation in maintaining postural stability among older adults with sensory deficits [J]. Front Public Health. 2023;11:116501010.3389/fpubh.2023.1165010 37213635 10.3389/fpubh.2023.1165010 PMC10194835

[37] Kolodziej M, Groll A, Nolte K, et al. Predictive modeling of lower extremity injury risk in male elite youth soccer players using least absolute shrinkage and selection operator regression [J]. Scand J Med Sci Sports. 2023;33(6):1021–33. 10.1111/sms.14322 36703247 10.1111/sms.14322

[38] De Blasiis P, Caravaggi P, FULLIN A, et al. Postural stability and plantar pressure parameters in healthy subjects: variability, correlation analysis and differences under open and closed eye conditions [J]. Front Bioeng Biotechnol. 2023;11:119812010.3389/fbioe.2023.1198120 37545891 10.3389/fbioe.2023.1198120 PMC10399229

[39] Nishimoto R, Fujiwar S, Kutoku Y, et al. Effect of dual-task interaction combining postural and visual perturbations on cortical activity and postural control ability [J]. NeuroImage. 2023;280:12035210.1016/j.neuroimage.2023.120352 37648121 10.1016/j.neuroimage.2023.120352

[40] Hall CD, Herdman SJ, Whitney SL, et al. Vestibular Rehabilitation for Peripheral vestibular hypofunction: an updated clinical practice Guideline from the Academy of neurologic physical therapy of the American Physical Therapy Association [J]. J Neurol Phys Ther. 2022;46(2):118–77. 10.1097/npt.0000000000000382 34864777 10.1097/NPT.0000000000000382 PMC8920012

[41] Maurus P, Jackson K, Cashaback JGA, et al. The nervous system tunes sensorimotor gains when reaching in variable mechanical environments [J]. iScience. 2023;26(6):106756. 10.1016/j.isci.2023.106756 37213228 10.1016/j.isci.2023.106756 PMC10197011

[42] Chancel M, Ehrsson HH. Proprioceptive uncertainty promotes the rubber hand illusion [J]. Cortex. 2023;165:70–85. 10.1016/j.cortex.2023.04.005 37269634 10.1016/j.cortex.2023.04.005 PMC10284257

[43] Yang F, Liu X. Relative importance of vision and proprioception in maintaining standing balance in people with multiple sclerosis [J]. Mult Scler Relat Disord. 2020;39:10190110.1016/j.msard.2019.101901 31918240 10.1016/j.msard.2019.101901

[44] Henry M, Baudry S. Age-related changes in leg proprioception: implications for postural control [J]. J Neurophysiol. 2019;122(2):525–38. 10.1152/jn.00067.2019 31166819 10.1152/jn.00067.2019 PMC6734411

[45] Raj MA, Bubnis MA. Knee meniscal tears [M]. StatPearls. Treasure Island (FL) companies. Disclosure: Matthew Bubnis declares no relevant financial relationships with ineligible companies.; StatPearls Publishing Copyright © 2023. StatPearls Publishing LLC; 2023. 28613721

[46] Harput G, Ulusoy B, Ozer H, et al. External supports improve knee performance in anterior cruciate ligament reconstructed individuals with higher kinesiophobia levels [J]. Knee. 2016;23(5):807–12. 10.1016/j.knee.2016.05.008 27460554 10.1016/j.knee.2016.05.008

[47] SáNCHEZ-HERáN Á, Agudo-Carmona D, FERRER-PEñA R, et al. Postural Stability in Osteoarthritis of the knee and hip: analysis of Association with Pain Catastrophizing and Fear-Avoidance beliefs [J]. Pm r. 2016;8(7):618–28. 10.1016/j.pmrj.2015.11.002 26578431 10.1016/j.pmrj.2015.11.002

[48] Hsu CJ, George SZ, Chmielewsk TL. Fear-avoidance and self-efficacy PSYCHOSOCIALpsychosocial factors are altered after partial meniscectomy and associated with rehabilitation outcomes [J]. Int J Sports Phys Ther. 2020;15(4):557–70. 33354389 PMC7735696

[49] Domenech J, Sanchis-alfonso V, LóPEZ L, et al. Influence of kinesiophobia and catastrophizing on pain and disability in anterior knee pain patients [J]. Knee Surg Sports Traumatol Arthrosc. 2013;21(7):1562–8. 10.1007/s00167-012-2238-5 23081711 10.1007/s00167-012-2238-5

[50] Roby C. Galen on the patient’s role in Pain diagnosis: sensation, Consensus, and metaphor [J]. Stud Anc Med. 2016;45:304–22. 26946683

[51] Hong H, Suh C, Namgung E, et al. Aberrant resting-state functional connectivity in Complex Regional Pain Syndrome: A Network-based Statistics Analysis [J]. Exp Neurobiol. 2023;32(2):110–8. 10.5607/en23003 37164651 10.5607/en23003 PMC10175954

[52] Gracel RH, Geisser ME, Giesecke T, et al. Pain catastrophizing and neural responses to pain among persons with fibromyalgia [J]. Brain. 2004;127(Pt 4):835–43. 10.1093/brain/awh098 14960499 10.1093/brain/awh098

[53] Kim HJ, Meeker TJ, Jung JY, et al. Biological sex influences psychological aspects of the biopsychosocial model related to chronic pain intensity and interference among South Korean patients with chronic secondary musculoskeletal pain in rheumatic diseases [J]. Front Psychol. 2023;14:106316410.3389/fpsyg.2023.1063164 37138999 10.3389/fpsyg.2023.1063164 PMC10150094

[54] George SZ, Dover GC, Wallace MR, et al. Biopsychosocial influence on exercise-induced delayed onset muscle soreness at the shoulder: pain catastrophizing and catechol-o-methyltransferase (COMT) diplotype predict pain ratings [J]. Clin J Pain. 2008;24(9):793–801. 10.1097/AJP.0b013e31817bcb65 18936597 10.1097/AJP.0b013e31817bcb65 PMC2669668

[55] Lethem J, Slade PD, Troup JD, et al. Outline of a fear-avoidance model of exaggerated pain perception–I [J]. Behav Res Ther. 1983;21(4):401–8. 10.1016/0005-7967(83)90009-8 6626110 10.1016/0005-7967(83)90009-8

[56] Rethman KK, Mansfield CJ, Moeller J, et al. Phys Ther. 2023;103(9). 10.1093/ptj/pzad074. Kinesiophobia Is Associated With Poor Function and Modifiable Through Interventions in People With Patellofemoral Pain: A Systematic Review With Individual Participant Data Correlation Meta-Analysis [J]. 10.1093/ptj/pzad074 PMC10517194 37354454

[57] Smith BE, Hendrick P, Smith TO, et al. Should exercises be painful in the management of chronic musculoskeletal pain? A systematic review and meta-analysis [J]. Br J Sports Med. 2017;51(23):1679–87. 10.1136/bjsports-2016-097383 28596288 10.1136/bjsports-2016-097383 PMC5739826

[58] Cai L, Liu Y, Xu H, et al. Incidence and risk factors of Kinesiophobia after total knee arthroplasty in Zhengzhou, China: a cross-sectional study [J]. J Arthroplasty. 2018;33(9):2858–62. 10.1016/j.arth.2018.04.028 29776855 10.1016/j.arth.2018.04.028

[59] Ericsson YB, Ringsberg K, Dahlberg LE. Self-efficacy, physical activity and health-related quality of life in middle-aged meniscectomy patients and controls [J]. Scand J Med Sci Sports. 2011;21(6):e150–8. 10.1111/j.1600-0838.2010.01201.x 22126722 10.1111/j.1600-0838.2010.01201.x

[60] Salles A. Self-efficacy as a measure of confidence [J]. JAMA Surg. 2017;152(5):506–7. 10.1001/jamasurg.2017.0035 28273296 10.1001/jamasurg.2017.0035

[61] Picha KJ, Jochimsen KN, Heebner NR, et al. Measurements of self-efficacy in musculoskeletal rehabilitation: a systematic review [J]. Musculoskelet Care. 2018;16(4):471–88. 10.1002/msc.1362 10.1002/msc.1362 PMC7944995 30238607

[62] Mcauley E, Blissmer B. Self-efficacy determinants and consequences of physical activity [J]. Exerc Sport Sci Rev. 2000;28(2):85–8. 10902091

[63] Wong EM, Chan SW, Chair SY. Effectiveness of an educational intervention on levels of pain, anxiety and self-efficacy for patients with musculoskeletal trauma [J]. J Adv Nurs. 2010;66(5):1120–31. 10.1111/j.1365-2648.2010.05273.x 20337801 10.1111/j.1365-2648.2010.05273.x

[64] Rini C, Porter LS, Somers TJ, et al. Automated internet-based pain coping skills training to manage osteoarthritis pain: a randomized controlled trial [J]. Pain. 2015;156(5):837–48. 10.1097/j.pain.0000000000000121 25734997 10.1097/j.pain.0000000000000121 PMC4402249

[65] Nwachukwu BU, Adjei J, RAUCK R C, et al. How much do psychological factors affect lack of Return to Play after Anterior Cruciate Ligament Reconstruction? A systematic review [J]. Orthop J Sports Med. 2019;7(5):2325967119845313. 10.1177/2325967119845313 31205965 10.1177/2325967119845313 PMC6537068

