# Supplementary material for: Current status and factors influencing kinesiophobia in patients with meniscus injury: a cross-sectional study
Source: J Orthop Surg Res. 2025 Jan 30;20:113. doi: 10.1186/s13018-025-05498-5 (PMC11780815; doi:10.1186/s13018-025-05498-5)
Supplement: Supplementary file 1 — Supplementary Material 1 [file 13018_2025_5498_MOESM1_ESM.docx]

**Clinical research trial Review Opinions of Fujian Provincial Hospital Ethics Committee**

**Lungren Scientific Research No. (K2023-03-041)**

| Review item | Project name | Efficacy and kinematic characteristics of acupotomy combined with platelet-rich plasma in the treatment of postoperative patients with meniscus injuries | | |
| --- | --- | --- | --- | --- |
|  | Topic origin | International Cooperation Projects □  National Research Projects □  Provincial Research Projects □  Provincial Health Commission Scientific Research Projects □  Hospital Research Projects □  Others ☒ | | |
|  | Project number | / | Starting and ending time | 2023.03-2024.03 |
|  | Department | Rehabilitation department II | Project Leader | Zhonghua Lin |
|  | Title | Chief physician | Contact phone number | 13774511377 |
| Admissibility review of documents | Ethics Application Form  Research Protocol  Informed Consent Form | | | |
| Review Methods | | Expedited review | | |
| Conclusion | In accordance with the Measures for Ethical Review of Biomedical Research Involving Human Beings (2016) of the Commission on Health and Family Planning (CHP), the Food and Drug Administration (FDA) Code of Practice for Quality Management of Drug Clinical Trials (2020), the Code of Practice for Quality Management of Clinical Trials of Medical Devices (2016), the Declaration of Helsinki (2013) of the World Medical Association (WMA), and the Committee for International Organizations of Medical Sciences (CIOMS) International Ethical Guidelines for Biomedical Research on Human Beings Guidelines for Human Biomedical Research (2002), the ethical principles were reviewed by this Ethics Committee and agreed to be conducted in accordance with the study protocol.  Whether the study was subjected to continuous review by the Ethics Committee during the course of the study ☒ Yes □ No  Frequency of review is from the date of study approval □ 6 months ☒12 months | | | |
| Ethics Committee (seal): | | | | |

March 28, 2023 Address: 134 East Street, Fuzhou City Zip Code: 350001 Tel: 0591-88216023
