# Supplementary figures and images for: Current status and factors influencing kinesiophobia in patients with meniscus injury: a cross-sectional study
Source: J Orthop Surg Res. 2025 Jan 30;20:113. doi: 10.1186/s13018-025-05498-5 (PMC11780815; doi:10.1186/s13018-025-05498-5)

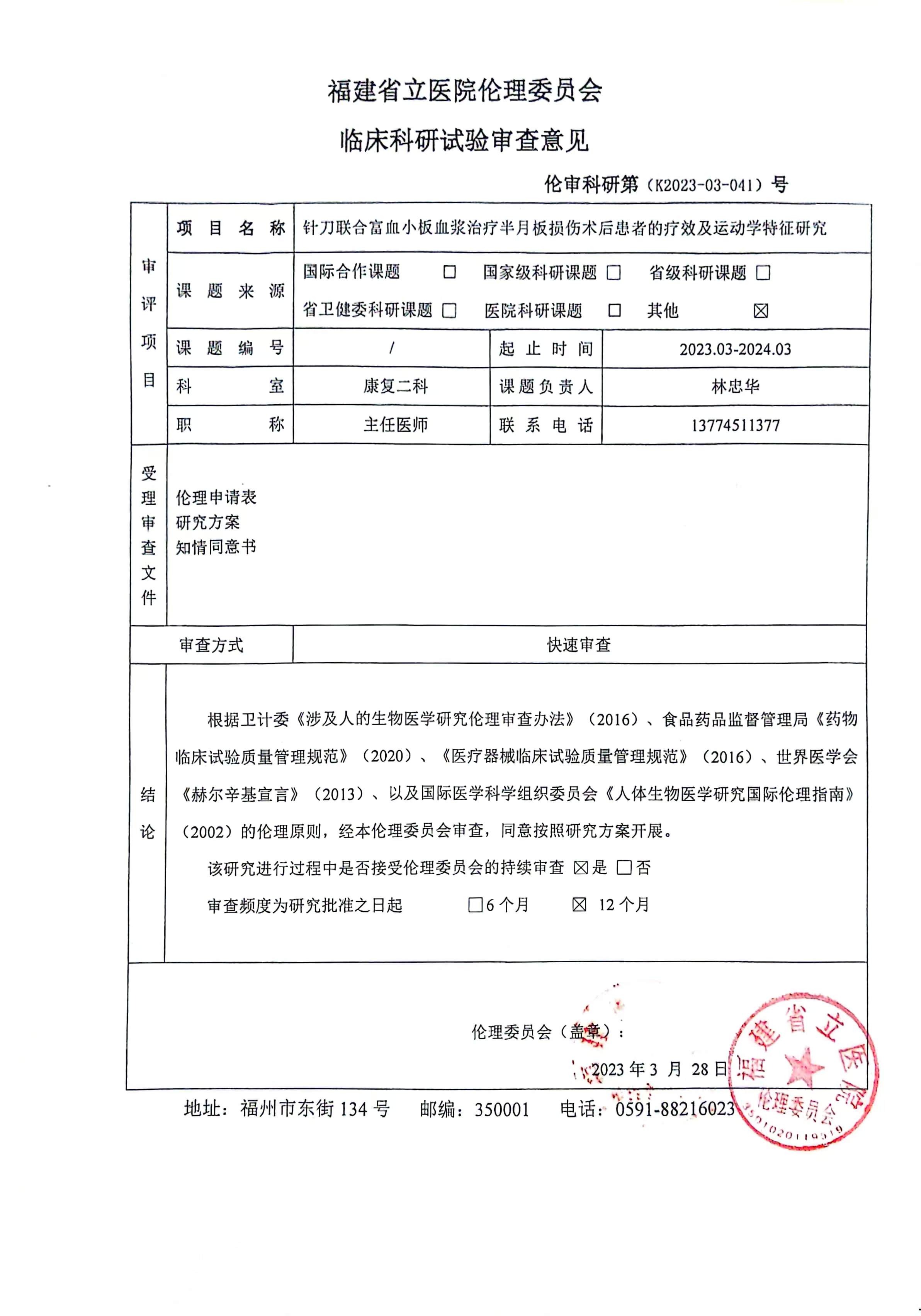

Supplement: Supplementary file 2 — Supplementary Material 2 [file 13018_2025_5498_MOESM2_ESM.doc]
